# Identification of 5-Gene Signature Improves Lung Adenocarcinoma Prognostic Stratification Based on Differential Expression Invasion Genes of Molecular Subtypes

**DOI:** 10.1155/2020/8832739

**Published:** 2020-12-31

**Authors:** Zhimin Zheng, Weijie Deng, Jiansheng Yang

**Affiliations:** ^1^Department of Thoracic Surgery, Jinjiang Municipal Hospital, Quanzhou, Fujian Province 362200, China; ^2^Department of Thoracic Surgery, The Second Clinical College of Fujian Medical University, Quanzhou, Fujian Province 362000, China

## Abstract

**Background:**

The acquisition of invasive tumor cell behavior is considered to be the cornerstone of the metastasis cascade. Thus, genetic markers associated with invasiveness can be stratified according to patient prognosis. In this study, we aimed to identify an invasive genetic trait and study its biological relevance in lung adenocarcinoma.

**Methods:**

250 TCGA patients with lung adenocarcinoma were used as the training set, and the remaining 250 TCGA patients, 500 ALL TCGA patients, 226 patients with GSE31210, 83 patients with GSE30219, and 127 patients with GSE50081 were used as the verification data sets. Subtype classification of all TCGA lung adenocarcinoma samples was based on invasion-associated genes using the R package ConsensusClusterPlus. Kaplan-Meier curves, LASSO (least absolute contraction and selection operator) method, and univariate and multivariate Cox analysis were used to develop a molecular model for predicting survival.

**Results:**

As a consequence, two molecular subtypes for LUAD were first identified from all TCGA all data sets which were significant on survival time. C1 subtype with poor prognosis has higher clinical characteristics of malignancy, higher mutation frequency of KRAS and TP53, and a lower expression of immune regulatory molecules. 2463 differentially expressed invasion genes between C1 and C2 subtypes were obtained, including 580 upregulation genes and 1883 downregulation genes. Functional enrichment analysis found that upregulated genes were associated with the development of tumor pathways, while downregulated genes were more associated with immunity. Furthermore, 5-invasion gene signature was constructed based on 2463 genes, which was validated in four data sets. This signature divided patients into high-risk and low-risk groups, and the LUDA survival rate of the high-risk group is significantly lower than that of the low-risk group. Multivariate Cox analysis revealed that this gene signature was an independent prognostic factor for LUDA. Compared with other existing models, our model has a higher AUC.

**Conclusion:**

In this study, two subtypes were identified. In addition, we developed a 5-gene signature prognostic risk model, which has a good AUC in the training set and independent validation set and is a model with independent clinical characteristics. Therefore, we recommend using this classifier as a molecular diagnostic test to assess the prognostic risk of patients with LUDA.

## 1. Introduction

Lung cancer is the leading cause of cancer-related deaths, both in China and globally. [1], with non-small-cell lung cancer (NSCLC) accounting for more than 80 percent of all lung cancers, and adenocarcinoma is the most common type of NSCLC. Many patients with newly diagnosed primary lung adenocarcinoma have developed distant metastases at the time of consultation. In the case of small early metastatic lesions, they cannot be detected by imaging in a timely manner, which makes accurate staging and timely treatment difficult [2]. For now, overall survival has improved with advances in detection technology and the availability of many targeted therapies and immunotherapies, but the 5-year survival rate for lung cancer patients after diagnosis is still less than 20% [3–5]. In lung cancer patients, organ failure and dysfunction associated with distant metastasis remain common causes of tumor-associated death [6]. In the choice of treatment for lung cancer patients, the patient's physical condition, treatment tolerance, and the presence of lymph nodes and distant metastasis should be taken into consideration to formulate a reasonable individualized treatment plan [7, 8].

Holistic gene expression profiling using microarray technology has proven to be an important tool to help reveal the molecular basis of cancer. The molecular classification of different cancers (e.g., colorectal cancer and lymphoma) consistently stratifies tumors into different subtypes, with prognostic outcomes independent of traditional clinical staging [9, 10]. Gene expression profiling of breast cancer [6] leads to subclassification of cancers previously thought to be homogeneous, allowing prediction of those most likely to benefit from chemotherapy [11] and overall survival [12]. Gene expression profiling has yielded many insights into the molecular basis of lung adenocarcinoma in the past decade [13–15]. However, this accumulation of knowledge has not yet provided clinical benefit in terms of improved patient treatment options or survival.

The aim of this study was to identify biomarkers of cancer cell invasion through a collection of invasive-specific gene signatures obtained from genome-wide gene expression profiles. Therefore, we recommend using this classifier as a molecular diagnostic test to assess the prognostic risk of patients with LUDA.

## 2. Material and Methods

### 2.1. Data Acquisition and Processing

**Figure 1:**
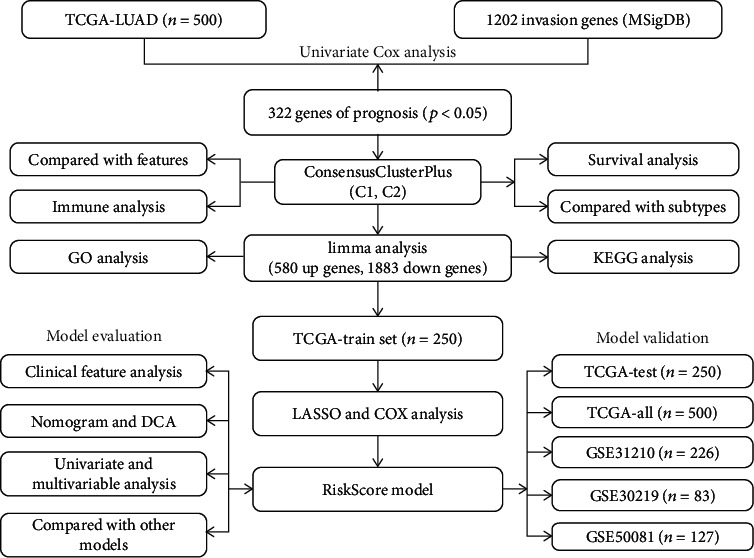
The workflow.

RNA-Seq data (FPKM) and clinical follow-up information data of lung adenocarcinoma (LUAD) were acquired from TCGA database (https://portal.gdc.cancer.gov/). The raw data of the three data sets GSE31210 [16], GSE30219 [17], and GSE50081 [18] were downloaded from the GEO database (https://www.ncbi.nlm.nih.gov/geo/); all three data sets were sequenced by GPL570 ([HG-U133_plus_2] Affymetrix Human Genome U133 Plus 2.0 Array). RMA (Robust Multiarray Average expression measure) in the R package affy (V1.66.0) [19] was used to process the expression profile data and normalized to obtain the expression profile. The metastasis-related gene sets were obtained from the 11 metastasis-related pathways in the c2.all.v7.0.symbols.gmt file on the GSEA [20] website (https://www.gsea-msigdb.org/gsea/index.jsp), and finally, a total of 1202 genes were obtained. All the enrolled samples have not undergone any treatment including chemotherapy and radiotherapy. The sample clinical information of the database is shown in Table S1. The work flow was showed in Figure 1.

### 2.2. Construction and Verification of Prognostic Models

First, the 500 samples in TCGA data set are divided into a training set and a validation set. In order to avoid random allocation deviations from affecting the stability of subsequent modeling, all samples are randomly grouped 100 times in advance with replacement according to the ratio of training set : validation set = 1 : 1. The training set and test set samples were tested using chi-squared test, and the results showed that our grouping had no preference. In the training data set, univariate cox survival analysis was performed for differentially expressed genes using the coxph function of the R package survival (V3.1-12), and *p* < 0.001 was selected as the threshold for filtering. Next, LASSO (least absolute shrinkage and selection operator) regression, multivariate Cox survival analysis, and stepAIC were conducted to further compress the filtered genes to reduce the number of genes in the risk model. The final selected genes were those of the prognostic model. The calculation formula of the prognostic risk model is as follows:

(1) $$\begin{array}{c} \text{RiskScore}=\overset{n}{\underset{i=1}{\sum }}\text{c}\text{oef}\left(i\right)*\text{gene}\left(i\right). \end{array}$$

Among them, coef(*i*) refers to the coefficient of the *i*th gene, and gene(*i*) refers to the expression level of the *i*th gene. Each sample is calculated to obtain a RiskScore value. The cutoff of the RiskScore is the middle value, those greater than the middle value are high-risk samples, and those less than or equal to the middle value are low-risk samples.

The same risk calculation method is verified on TCGA training data set, all TCGA data sets, and three completely independent data sets GSE31210, GSE30219, and GSE50081. At the same time, we draw the ROC curves of RiskScore at different time points on and calculate the corresponding AUC values to judge the performance of the model.

### 2.3. Statistic and Analysis

Univariate cox analysis was used to analyzed 1202 metastasis-related genes using the coxph function of the R package survival (V3.1-12), and the genes related to the prognosis of lung adenocarcinoma (*p* < 0.05) were obtained. Next, the R package ConsensusClusterPlus (V1.48.0; parameters: reps = 100, pItem = 0.8, pFeature = 1, distance = “Pearson”) was used to uniformly cluster TCGA samples (D2 and Euclidean distance are used as the clustering algorithm and distance measure). The chi-squared test was used to identify the distribution of clinical features on molecular subtypes. The R software package MCPcounter (V1.2.0) was used to calculate the immune cell score of each sample. The R software package limma (V3.44.3) was used to perform differential expression gene between molecular subtypes, and FDR < 0.001 and ∣FC | >1.5 acted as thresholds. R package clusterProfiler (V3.16.0) was used to perform GO function annotation and KEGG pathway enrichment analysis on differentially expressed genes, and FDR < 0.05 as the threshold. Univariate and multivariate Cox survival analyses of clinical characteristics and RiskScore were used to demonstrate the independence of the RiskScore model. Based on the results of univariate and multivariate Cox survival analyses, the nomogram and the correction chart of the nomogram were constructed to provide the basis for clinical diagnosis and prognosis. And at the same time, we drew a DCA diagram to prove the reliability of the model.

## 3. Results

### 3.1. Identification of Molecular Subtypes

**Figure 2:**
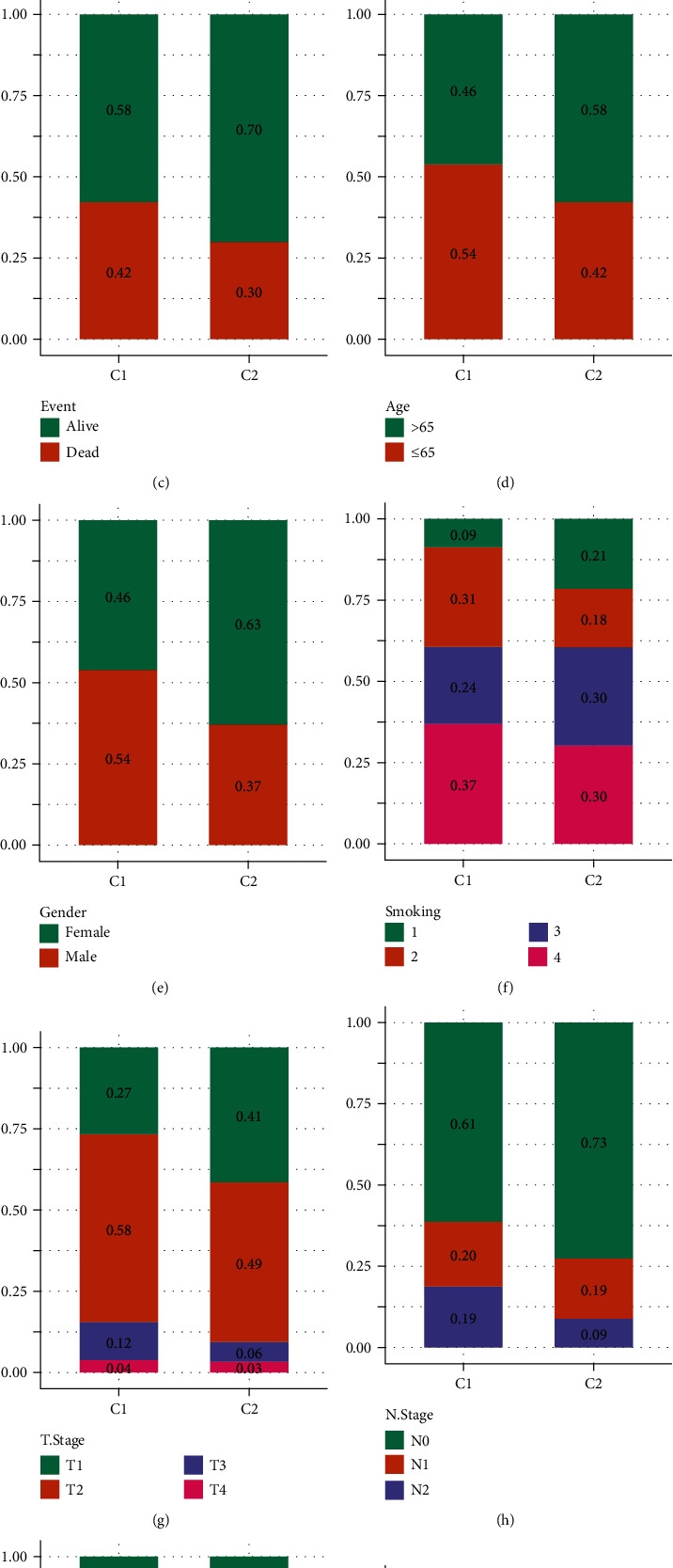
Identification of molecular subtype: (a) consistent cluster analysis (*K* = 2); (b) KM curves between molecular subtypes; (c) comparison of survival status distributions between molecular subtypes; (d) comparison of age distributions between molecular subtypes; (e) comparison of gender distributions between molecular subtypes; (f) comparison of smoking status distributions between molecular subtypes; (g) comparison of T stage distributions between molecular subtypes; (h) comparison of N stage distributions between molecular subtypes; (i) comparison of M staging distributions between molecular subtypes; (j) comparison of stage staging distributions between molecular subtypes.

Univariate cox survival analysis of 1130 metastasis-related genes was performed using the coxph function of the R package survival, and 322 genes related to the prognosis of LUDA were obtained (*p* < 0.05). The R package ConsensusClusterPlus was used to cluster TCGA samples uniformly and divide them into two categories (called C1 and C2, respectively) using 322 genes (Figure 2(a)). The KM survival curve between molecular subtypes shows that the prognosis of molecular subtype C2 is better than that of C1 (Figure 2(b), *p* = 0.00038). We compared the distribution of survival status, gender, age, T stage, N stage, M stage, stage, and smoking status among the two subtypes. The results showed that the number of dead, male, smoking sample was higher, while the T1 samples, N0 samples, and stage I samples are lower in the C1 subtype, compared with the C2 subtype (Figures 2(c)–2(j)). The distribution data of the above clinical characteristics indicated that the C1 subtype has a worse prognosis.

### 3.2. Analysis of Mutational Molecular Events, Existing Subtypes, and Immunity between Molecular Subtypes

**Figure 3:**
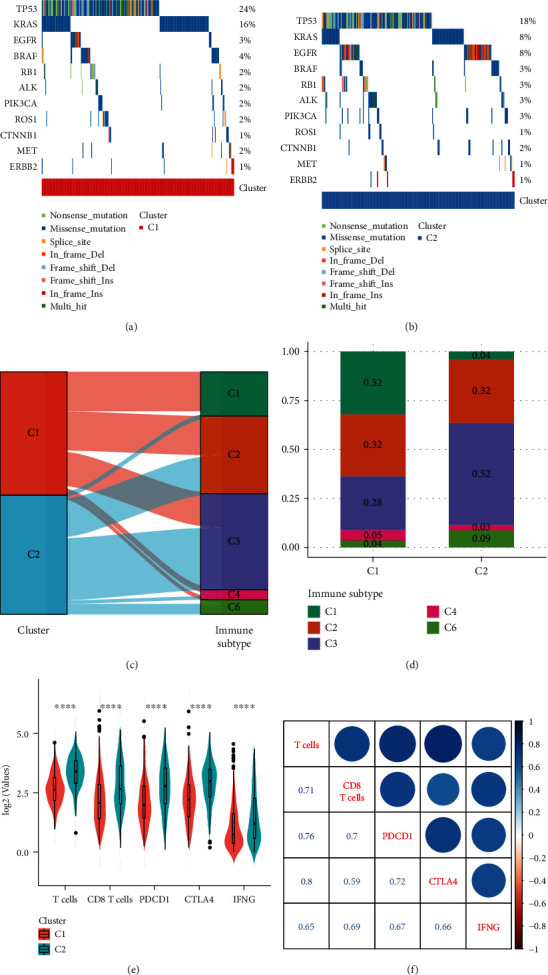
Analysis of mutational molecular events, existing subtypes, and immunity between molecular subtypes: (a) map of key gene mutations of molecular subtype C1; (b) map of key gene mutations of molecular subtype C2; (c) mulberry diagram of molecular subtypes and existing molecular subtypes; (d) distribution of existing molecular subtypes in our molecular subtypes; (e) comparison of immune score and immune checkpoint gene expression among molecular subtypes; (f) correlation between immune cell score and immune-related genes.

The SNV/InDel detected by MUTect was downloaded from TCGA database, and the mutation map of key mutated genes in LUDA such as EGFR, KRAS, TP53, and BRAF was selected. The mutation map of the key mutant genes in the C1 subtype showed that the mutation frequency of KRAS and TP53 in the C1 subtype was higher than that in the C2 subtype, while the mutation frequency of the EGFR in the C1 subtype was lower than that in the C2 subtype (Figures 3(a) and 3(b)). The six published immunoinfiltrating molecular subtypes were further compared with the molecular subtypes we found; most LUDA patients in TCGA data belong to the C1, C2, and C3 immune subtypes (about 89.8%), of which the C3 immune subtype has the best prognosis (Figure S1). Interestingly, the C3 immune subtype samples mostly overlap with our C2 subtype samples (Figures 3(c) and 3(d)), which is consistent with the good prognosis of our C2 subtype.

To identify the relationship between the immune cell scores in the two molecular subtypes, the R software package MCPcounter was used to calculate the immune cell scores (B lineage, cytotoxic lymphocytes, endothelial cells, fibroblasts, monocytic lineage, myeloid dendritic cells, and neutrophils) of each sample. The results showed that except for fibroblasts, the scores of other immune cells are higher in the C2 subtype than that in the C1 subtype, which includes T cells and CD8 T cells (Figure S2). It may also be a reason for the better prognosis of the C2 subtype.

In recent years, immune checkpoint suppression (ICI) research has made breakthroughs in the clinical response of a variety of human cancers. However, most cancer patients do not benefit from ICI. We compared the expression of PDCD1 (PD-L1), CTLA4, and IFNG (IFN-*γ*) genes in molecular subtypes and found that the expression of these three genes in the C2 subtype was significantly higher than that in the C1 subtype (Figure 3(e)). In addition, we calculated the Pearson correlation between PDCD1, CTLA4, and IFNG gene expression and the immune cell scores of T cells and CD8 T cells and found that there is a strong positive correlation between them (Figure 3(f)). The above results indicated that molecular subtype C2 may have a better response to immunotherapy.

### 3.3. Identification of Differentially Expressed Genes

**Figure 4:**
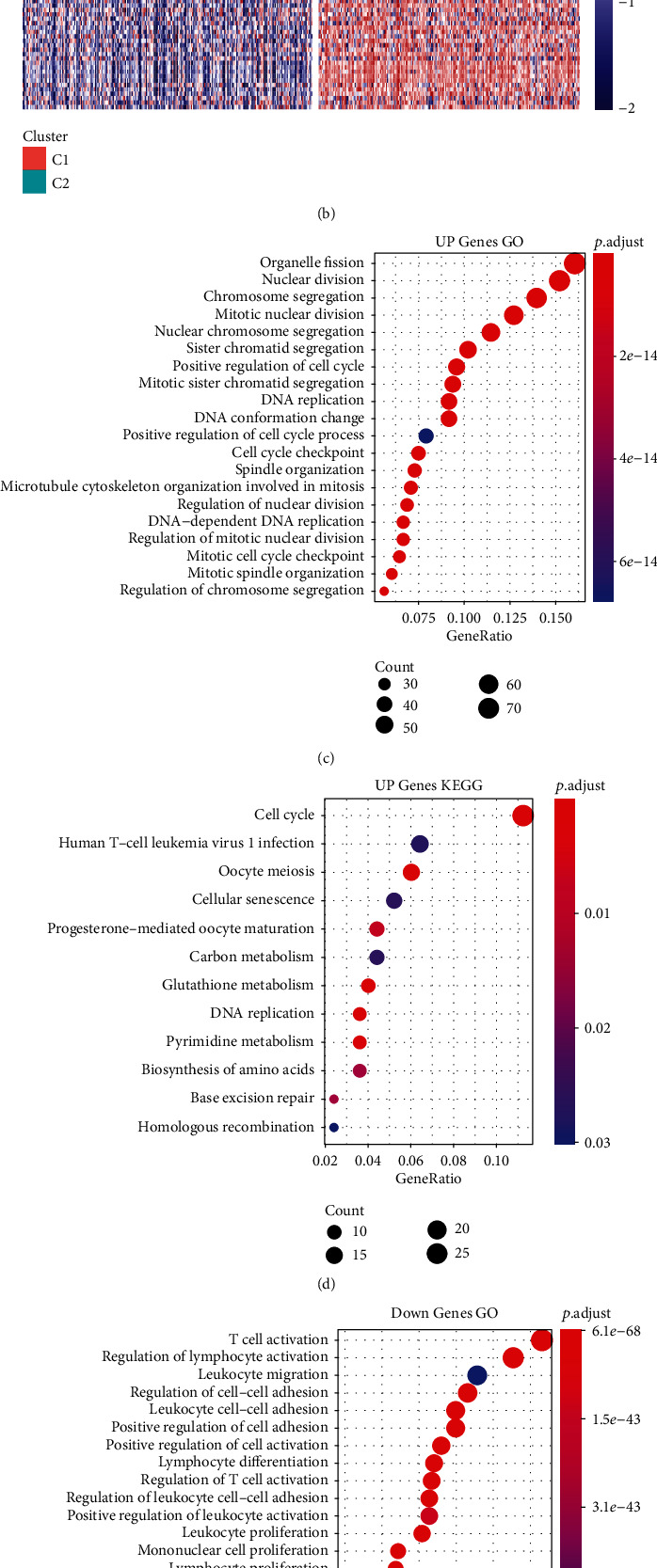
Identification of differentially expressed genes: (a) volcanic map of genes with differentially expressed genes between C1 and C2 molecular subtypes; (b) heat map of differentially expressed genes between C1 and C2 molecular subtypes; (c, d) GO and KEGG analysis in upregulation genes; (e, f) GO and KEGG analysis in downregulation genes.

Through the limma package, a total of 2463 differentially expressed genes are filtered, of which 580 are upregulated and 1883 are downregulated (Figure 4(a)). 100 genes with the largest differential upregulation and downregulation were selected to draw a heat map (Figure 4(b)). GO functional enrichment analysis and KEGG pathway analysis of differentially expressed genes were performed using the R software package WebGestaltR (V0.4.2). 580 upregulated differential genes were annotated to 320 functions with significant differences such as cell replication, nuclear division related to mitosis, DNA replication, DNA-dependent DNA replication, and regulation of mitotic nuclear division (Figure 4(c)). 580 upregulated genes were annotated to 12 significant KEGG pathways, including cell cycle, DNA replication, base excision repair, homologous recombination, and other tumor-related pathways (Figure 4(d)). 1883 downregulated genes were annotated to 1109 function terms with significant differences, containing immune-related T cell activation, regulation of lymphocyte activation, regulation of T cell activation, and positive regulation of T cell activation (Figure 4(e)). 1883 downregulated expression genes were annotated to 61 significant KEGG pathways, including Th1 and Th2 cell differentiation, chemokine signaling pathway, cytokine-cytokine receptor interaction, natural killer cell-mediated cytotoxicity, T cell receptor signaling pathway, B cell receptor signaling pathway, and other immune-related pathways (Figure 4(f)).

### 3.4. Construction and Verification of Prognostic Models Based on Differential Genes of Molecular Subtypes

**Figure 5:**
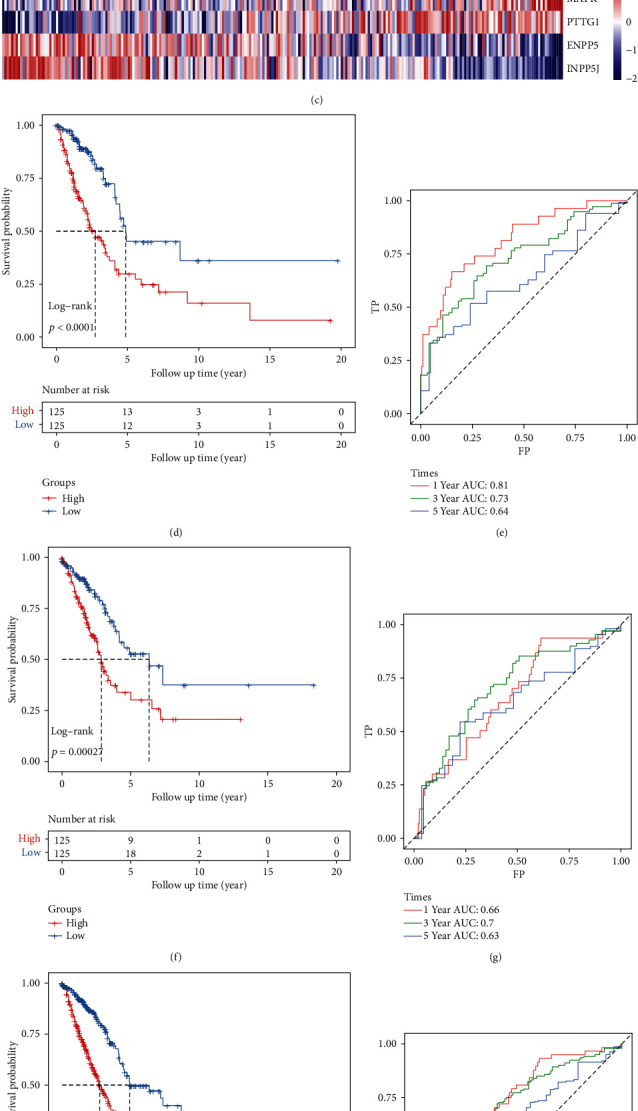
Construction and verification of prognostic models based on differential genes of molecular subtypes: (a, b) the distribution of RiskScore and the corresponding distribution of survival state in TCGA training data set; (c) heat map of gene expression of the RiskScore model; (d, e) KM curve and ROC curve of the high- and low-risk group in TCGA training data set; (f, g) KM curve and ROC curve of the high- and low-risk group in TCGA validation data set; (h, i) KM curve and ROC curve of the high- and low-risk group in all TCGA data sets.

On the training data set, univariate Cox survival analysis was performed on the 2463 differential expression genes, and 52 prognostic-related genes (*p* < 0.001) were obtained. Then, the R software package glmnet was used to perform LASSO cox regression analysis. First, the change trajectory of each independent variable is analyzed (Figure S3A), from which it can be seen that with the gradual increase of lambda, the number of independent variable coefficients approaching 0 also increases gradually. The 5-fold cross-validation was used to build the model, and the confidence interval under each lambda was analyzed (Figure S3B); when lambda = 0.02797, the model reached the optimal value. For this reason, 12 genes were selected at lambda = 0.02797 for the next step of analysis. These 12 genes were subjected to multivariate Cox survival analysis and the stepAIC method to further reduce the number of genes. Finally, 5 genes were used to construct the model (Table 1). The expression of these five genes made a significant prognosis difference between the risk of high and low expression in the sample (Figure S4). The RiskScore of each sample in TCGA training data set was obtained according to the RiskScore calculation formula, and then, median value was used as the cutoff point. If the RiskScore is greater than the median value, it is high risk, and if the RiskScore is less than or equal to the median value, it is low risk (Figure 5(a)). The survival time distribution of TCGA training set samples from low risk to high risk is plotted, and in the low-risk area, the proportion of survivors is higher (Figure 5(b)). Higher expressions of KRT8, MAFK, and PTTG1 were positively correlated with the risk score, and the three were therefore considered as risk factors; Lower expressions of ENPP5 and INPP5J were negatively correlated with the risk score, and were regarded as protective factors (Figure 5(c)). KM survival curve analysis found that the high-risk group and the low-risk group had significant prognostic differences (Figure 5(d), *p* < 0.0001). The ROC curve analysis showed that the 1, 3, and 5-year AUC of RiskScore were 0.64, 0.73, and 0.81, respectively (Figure 5(e)).

In order to verify the reliability of our risk model, we used TCGA validation data set and all data sets for verification. The results showed that our risk model in TCGA validation data set (Figures 5(f) and 5(g)) and all data sets (Figures 5(h) and 5(i)) also have good results.

### 3.5. Robustness of the Model

**Figure 6:**
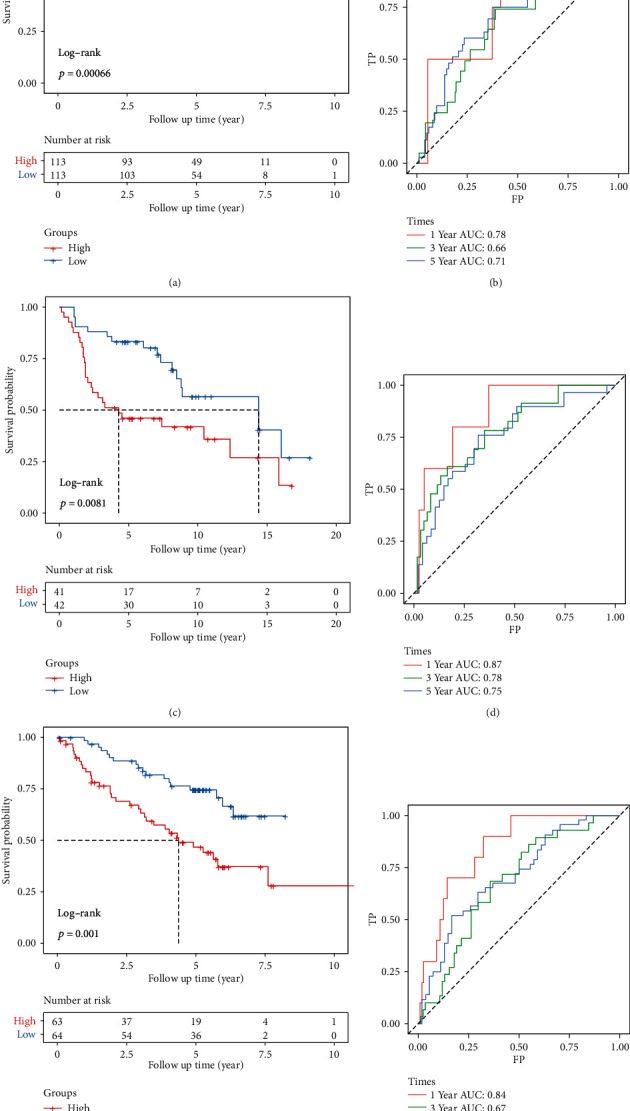
Robustness of the model: (a, b) KM curve and ROC curve of the high- and low-risk group in the GSE31210 data set; (c, d) KM curve and ROC curve of the high- and low-risk group in the GSE30219 data set; (e, f) KM curve and ROC curve of the high- and low-risk group in the GSE50081 data set.

At the same time, in order to further verify that our risk model has good effects on different platforms and different data sets, the risk model was verified in three independent data sets GSE31210, GSE30219, and GSE50081. We used the same risk coefficient to calculate the risk score of the sample in each data set and divide the sample into high-/low-risk groups with the median cutoff. We found that the KM curves of the high- and low-risk groups of the three data sets have significant differences (Figures 6(a), 6(c), and 6(e)). The ROC curves of RiskScore in three data sets all have higher AUC (Figures 6(b), 6(d), and 6(f)). This proved that our model has good performance and versatility.

### 3.6. Analysis of Risk Score on Clinical Characteristics

**Figure 7:**
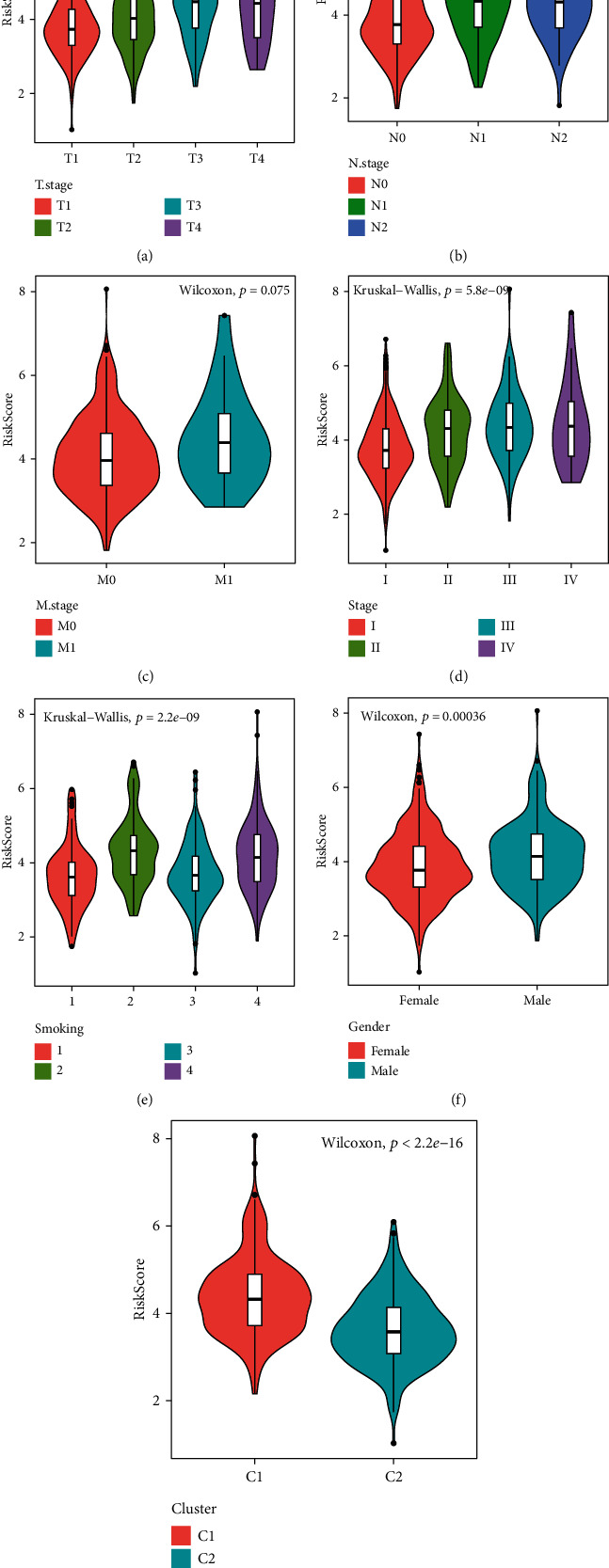
Analysis of risk score on clinical characteristics: (a) the comparison of RiskScore among T1-T4 stage samples; (b) the comparison between RiskScore among N0-N2 stage samples; (c) the comparison between RiskScore among M0-M1 stage samples; (d) the comparison between RiskScore in the stage I-stage IV samples; (e) the comparison between RiskScore among samples of smoking; (f) the comparison between RiskScore in gender (male and female) samples; (g) the comparison between RiskScore among molecular subtypes C1 and C2.

The distribution of RiskScore among clinical feature groups showed significant differences between T Stage, N Stage, stage, smoking, gender, and our molecular subtype (*p* < 0.05) (Figure 7). In our molecular subtypes, the risk score of the worse-prognosis C1 subtype is significantly higher than that of the C2 subtype with better prognosis.

Furthermore, we compared the differences of our models in the chemotherapy and radiotherapy samples, and the results are shown in Figure S5. Our models showed significant differences in the chemotherapy samples, while there was no significant difference in the radiotherapy samples.

### 3.7. Univariate and Multivariate Survival Analysis

In order to identify the independence of the RiskScore model in clinical application, we analyzed the relevant HR, 95% CI of HR, and *p* value in the clinical information of the entire TCGA data using univariate and multivariate survival analysis. We systematically analyzed the clinical information of TCGA patient records, including age, gender, T stage, N stage, M stage, smoking, stage, and our RiskType grouping information (Table 2). The results found that in both univariate and multivariate survival analyses, RiskType was significant in prognosis, which shows the independent reliability of our model. At the same time, the clinical features of T stage and N stage are also significant in univariate and multivariate survival analysis, and they are also independent prognostic factors.

### 3.8. Nomogram and Forest Diagram Constructed by RiskScore and Clinical Features

**Figure 8:**
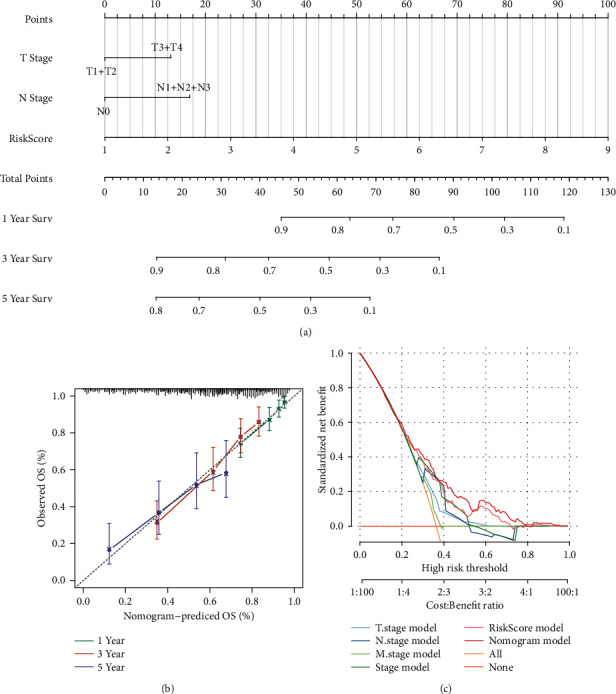
Nomogram and forest diagram constructed by RiskScore and clinical features: (a) a nomogram model was built based on the independent prognostic factors T stage, N stage, and RiskScore in all TCGA data sets; (b) calibration chart of the nomogram; (c) DCA diagram of clinical features and the RiskScore.

We built a nomogram model based on the independent prognostic factors T stage, N stage, and RiskScore on all TCGA data sets. From the model results, the RiskScore feature has the greatest impact on survival prediction, indicating that the risk model could predict prognosis better (Figure 8(a)). At the same time, we corrected the nomogram (1-, 3-, and 5-year data) to visualize the performance of the nomogram (Figure 8(b)). DCA (Decision Curve Analysis) is a simple method to evaluate clinical prediction models, diagnostic tests, and molecular markers. DCA curve analysis showed that RiskScore has better results, and the model combined with clinical features (nomogram model) has better results (Figure 8(c)).

### 3.9. Advantages of the Risk Model

**Figure 9:**
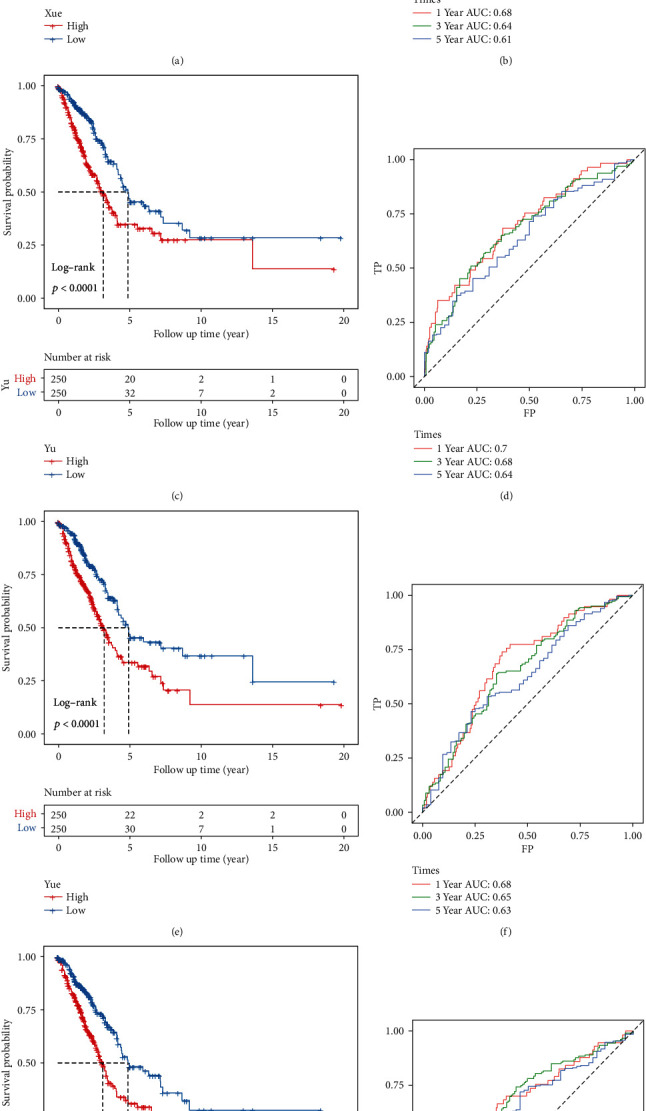
Advantages of the risk model: (a, b) KM curve of the high/low group samples and the ROC curve of the 12-gene risk model (Xue); (c, d) KM curve of the high/low group samples and the ROC curve of the 5-gene model (Yu); (e, f) KM curve of the high/low grouped samples and the ROC curve of the 3-gene model (Yue); (g, h) KM curve of the high/low group samples and the ROC curve of the 10-gene model (Mao).

Four prognostic-related risk models (12-gene model (Xue) [21], 5-gene model (Yu) [22], 3-gene model (Yue) [23], and 10-gene model (Mao) [24]) were selected to compare with our 5-gene model. In order to make the models comparable, we used the same method to calculate the RiskScore of each LUAD sample in TCGA based on the corresponding genes in these four models. The KM survival curve showed that the LUAD prognosis of the high and low group samples of the four models is also different (Figures 9(a), 9(c), 9(e), and 9(g)). However, the 1-, 3-, and 5-year AUC values of the four models on TCGA data are all lower than those of our model (Figures 9(b), 9(d), 9(f), and 9(h)), implying that our model has better performance.

## 4. Discussion

With the development of microarray technology and RNA sequencing technology, many studies have used gene expression profiles to classify tumors [25, 26]. Gene expression profiles have been used to divide LUAD into subgroups. For example, Bhattacharjee et al. used hierarchical and probabilistic clustering methods to define different subtypes of LUAD [27], showing the ability of gene expression profiling to assist LUAD diagnosis. The hierarchical clustering method was used to identify the expression patterns of 835 specific genes for lung cancer subtypes [28]. Similarly, Hayes *et al*. and Wilkerson *et al*. used ConsensusClusterPlus to determine the subtype of LUAD using gene expression data [29, 30]. Chen *et al*. combined multiplatform genomics data sets, including DNA methylation, DNA copy changes, mRNA expression, miRNA expression, and protein expression and proposed a “cluster-cluster” lung cancer classification analysis method [31]. Hu *et al*. used genome-wide mRNA expression profile to establish the robust molecular subtypes of LUAD by using a combination method [32]. Many studies have also found different LUAD subtypes, which have different immune infiltration characteristics and molecular mechanisms [33, 34]. More detailed genetic classification of tumors may be more effective for clinical precision therapy. However, the molecular subtype identification of metastasis-related genes in LUAD is still unclear. In our work, two molecular subtypes of LUAD were established using metastasis-related mRNA expression profile. Furthermore, the survival analysis showed that patients in subtype C2 had the best survival rate. By comparing the clinical characteristics of molecular subtypes, the clinical characteristics of subtype C2 samples with good prognosis are in the early stage of tumor. The mutation frequency of KRAS and TP53 of the C2 subtype is significantly lower, and the immune cell score is higher, compared with C1. These analysis results showed the reliability of our molecular subtype and the reason for the better prognosis of the C2 subtype.

Additionally, a metastasis-specific 5-mRNA signature was derived based on differentially expressed genes between C1 and C2, containing KRT8, MAFK, PTTG1, ENPP5, and INPP5J, which identified groups with low and high risk in terms of TCGA training data set. KRT8 is a type II basic intermediate filament (IF) protein, associated with EMT, which is essential for the occurrence and metastasis of various cancers. KRT8 mRNA expression was significantly upregulated in LUAD tissues, indicating unfavorable prognosis for poor OS and RFS in LUAD patients [35, 36]. Overexpression of MAFK induced epithelial-mesenchymal transition (EMT) phenotypes and promoted triple-negative breast cancer formation and invasion in mice [37]. Pituitary tumor transforming gene 1 (PTTG1) is highly expressed in many tumors and regulates tumor growth and progression. The expression of PTTG1 protein was markedly upregulated in LUAC tissues and was positively associated with the lymphatic invasion of the tumor [38]. INPP5J protein expression was drastically decreased in human ovarian cancer cells [39] and acted as a vital negative regulator of PI3K/Akt signaling in numerous types of human cancers [40]. There are few studies on ENPP5 in tumors. For the first time, we reported a metastasis gene signature identified using bioinformatics methods in LUDA patients, which displays prognostic value for patients.

However, some limitations of the current study should be considered. First, the population ethnicity in TCGA database is primarily limited to whites and blacks, and extrapolation of the findings to other ethnicities is needed. Second, the prognostic model needs to be further validated in multicenter clinical trials and prospective studies. In the future, we will also explore the possibility of including additional prognostic variables to further improve performance. Other regression modeling approaches will be applied to determine if predictive accuracy can be further improved. Basic experimental studies are also a limitation of our study.

We first identified a new 5-gene marker metastasis risk model that performed well in predicting the prognosis of LUAD. These 5 genes have complex molecular functions, among which ENPP5 and INPP5J have not been reported to be related to LUAD. Our study emphasized the relationship between metastasis-related genes and the prognosis of LUAD. Our results may provide precision and personalized treatment for clinical lung adenocarcinoma patients.

## Figures and Tables

**Table 1:** The detailed information of five prognostic mRNAs significantly associated with overall survival in patients with LUAD.

Gene	Coef	HR	HR (lower, 0.95)	HR (upper, 0.95)	*p*
KRT8	0.258	1.295	1.000	1.675	4.97*E*-02
MAFK	0.365	1.441	1.182	1.756	3.01*E*-04
PTTG1	0.355	1.426	1.136	1.792	0.002
ENPP5	-0.235	0.791	0.649	0.964	0.020
INPP5J	-0.434	0.648	0.496	0.846	0.001

**Table 2:** Univariate and multivariate Cox survival analysis.

Feature	Univariable analysis				Multivariable analysis			
	HR	95% CI of HR		*p*	HR	95% CI of HR		*p*
		Lower	Upper			Lower	Upper	
Age								
≤65				0.192				0.068
>65	1.217	0.906	1.635		1.406	0.976	2.025	
Gender								
Female				0.747				0.813
Male	1.049	0.784	1.405		0.957	0.667	1.375	
T stage								
T1-T2				<1*e*-5				0.008
T3-T4	2.298	1.568	3.366		1.934	1.19	3.143	
N stage								
N0				<1*e*-5				0.002
N1-N3	2.58	1.918	3.47		1.986	1.292	3.054	
M stage								
M0				0.006				0.226
M1	2.133	1.245	3.654		1.515	0.773	2.967	
Smoking								
1				0.536				0.541
2-4	0.878	0.581	1.326		0.852	0.51	1.423	
Stage								
I+II				<1*e*-5				0.678
III+IV	2.584	1.893	3.527		1.118	0.66	1.892	
RiskType								
Low				<1*e*-5				<1*e*-5
High	2.497	1.834	3.4		2.165	1.461	3.209	

## Data Availability

The analyzed data sets generated during the study are available from the corresponding author on reasonable request.

## Supplementary Materials

Supplementary Materials Figure S1: KM survival curve of the six published immunoinfiltrating molecular subtypes. Figure S2: immune cell scores (B lineage, cytotoxic lymphocytes, endothelial cells, fibroblasts, monocytic lineage, myeloid dendritic cells, and neutrophils) of each sample. Figure S3: A: with the gradual increase of lambda, the number of independent variable coefficients approaching 0 also increases gradually. B: when lambda = 0.02797, the model reached the optimal value. Figure S4: the expression of five genes made a significant prognosis difference between the risk of high and low expression in the sample. Figure S5: the differences of our models in the chemotherapy and radiotherapy samples. Table S1: The sample clinical information of databases.
