# Supplementary material for: Identification of 5-Gene Signature Improves Lung Adenocarcinoma Prognostic Stratification Based on Differential Expression Invasion Genes of Molecular Subtypes
Source: Biomed Res Int. 2020 Dec 31;2020:8832739. doi: 10.1155/2020/8832739 (PMC7790577; doi:10.1155/2020/8832739)
Supplement: Supplementary Materials — Figure S1: KM survival curve of the six published immunoinfiltrating molecular subtypes. Figure S2: immune cell scores (B lineage, cytotoxic lymphocytes, endothelial cells, fibroblasts, monocytic lineage, myeloid dendritic cells, and neutrophils) of each sample. Figure S3: A: with the gradual increase of lambda, the number of independent variable coefficients approaching 0 also increases gradually. B: when lambda = 0.02797, the model reached the optimal value. Figure S4: the expression of five genes made a significant prognosis difference between the risk of high and low expression in the sample. Figure S5: the differences of our models in the chemotherapy and radiotherapy samples. Table S1: The sample clinical information of databases. [file 8832739.f1.zip › Figure S4.pdf]

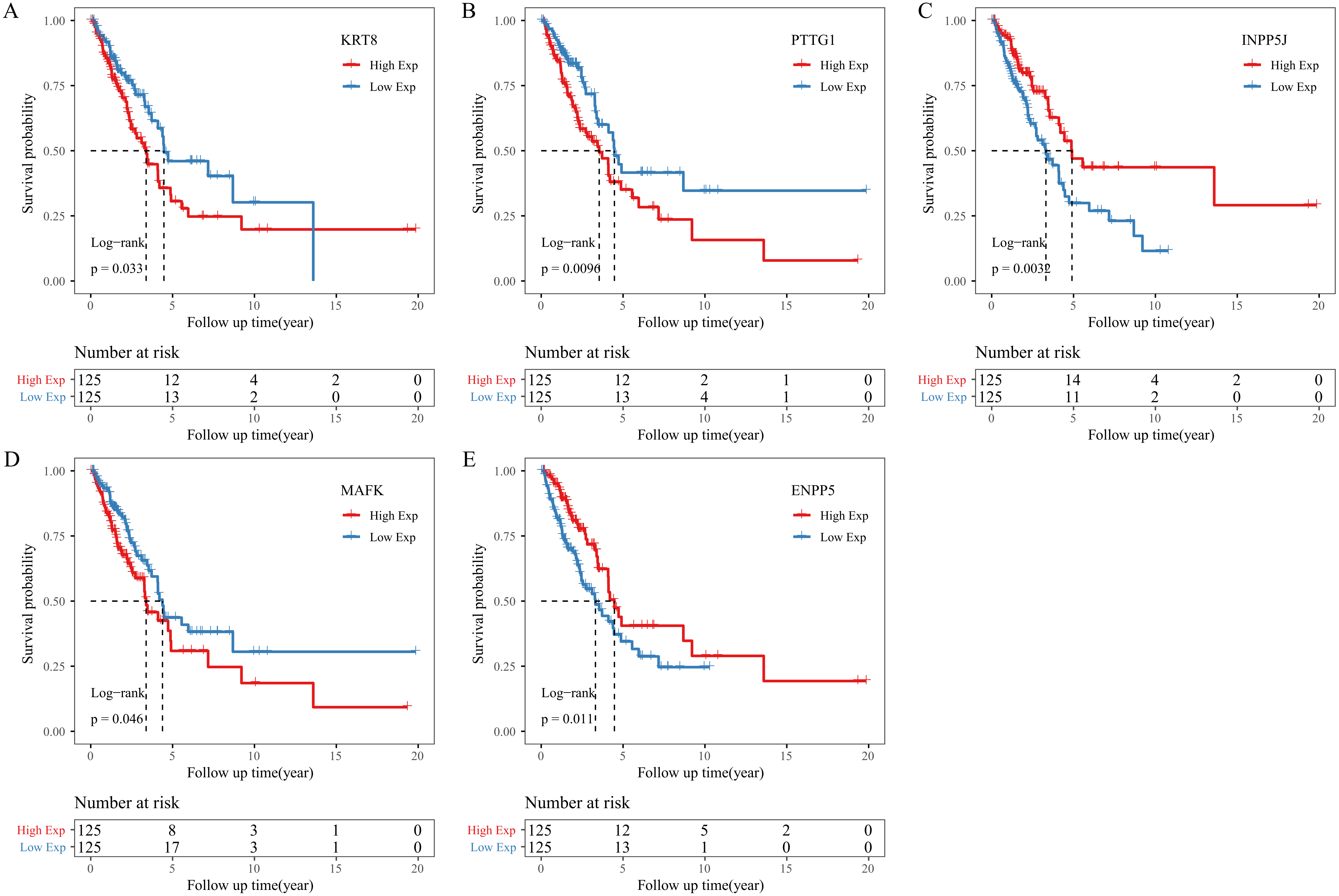
A
KRT8
High Exp
Low Exp
Survival probability
1.00
0.75
0.50
0.25
0.00
Log−rank
p = 0.033
0
5
10
15
20
Follow up time(year)
Number at risk
High Exp 125 12 4 2 0
Low Exp 125 13 2 0 0
Follow up time(year)
B
PTTG1
High Exp
Low Exp
Survival probability
Log−rank
p = 0.0096
Follow up time(year)
Number at risk
High Exp 125 12 2 1 0
Low Exp 125 13 4 1 0
Follow up time(year)
C
INPP5J
High Exp
Low Exp
Survival probability
Log−rank
p = 0.0032
Follow up time(year)
Number at risk
High Exp 125 14 4 2 0
Low Exp 125 11 2 0 0
Follow up time(year)
D
MAFK
High Exp
Low Exp
Survival probability
Log−rank
p = 0.046
Follow up time(year)
Number at risk
High Exp 125 8 3 1 0
Low Exp 125 17 3 1 0
Follow up time(year)
E
ENPP5
High Exp
Low Exp
Survival probability
Log−rank
p = 0.011
Follow up time(year)
Number at risk
High Exp 125 12 5 2 0
Low Exp 125 13 1 0 0
Follow up time(year)
