# Supplementary material for: Identification of 5-Gene Signature Improves Lung Adenocarcinoma Prognostic Stratification Based on Differential Expression Invasion Genes of Molecular Subtypes
Source: Biomed Res Int. 2020 Dec 31;2020:8832739. doi: 10.1155/2020/8832739 (PMC7790577; doi:10.1155/2020/8832739)
Supplement: Supplementary Materials — Figure S1: KM survival curve of the six published immunoinfiltrating molecular subtypes. Figure S2: immune cell scores (B lineage, cytotoxic lymphocytes, endothelial cells, fibroblasts, monocytic lineage, myeloid dendritic cells, and neutrophils) of each sample. Figure S3: A: with the gradual increase of lambda, the number of independent variable coefficients approaching 0 also increases gradually. B: when lambda = 0.02797, the model reached the optimal value. Figure S4: the expression of five genes made a significant prognosis difference between the risk of high and low expression in the sample. Figure S5: the differences of our models in the chemotherapy and radiotherapy samples. Table S1: The sample clinical information of databases. [file 8832739.f1.zip › Supplementary figure legens.docx]

**Supplementary figure legends**

**Supplementary Figure 1**

KM curve of existing immune subtypes.

**Supplementary Figure 2**

Comparison of immune cell scores in molecular subtypes.

**Supplementary Figure 3**

LASSO regression analysis trajectory. A: The trajectory of each independent variable, the log of lambda on the horizontal axis and the coefficient on the vertical axis. B: Confidence intervals for each lambda.

**Supplementary Figure 4**

KM curves for genes in risk models.
