# Supplementary figures and images for: Identification of 5-Gene Signature Improves Lung Adenocarcinoma Prognostic Stratification Based on Differential Expression Invasion Genes of Molecular Subtypes
Source: Biomed Res Int. 2020 Dec 31;2020:8832739. doi: 10.1155/2020/8832739 (PMC7790577; doi:10.1155/2020/8832739)

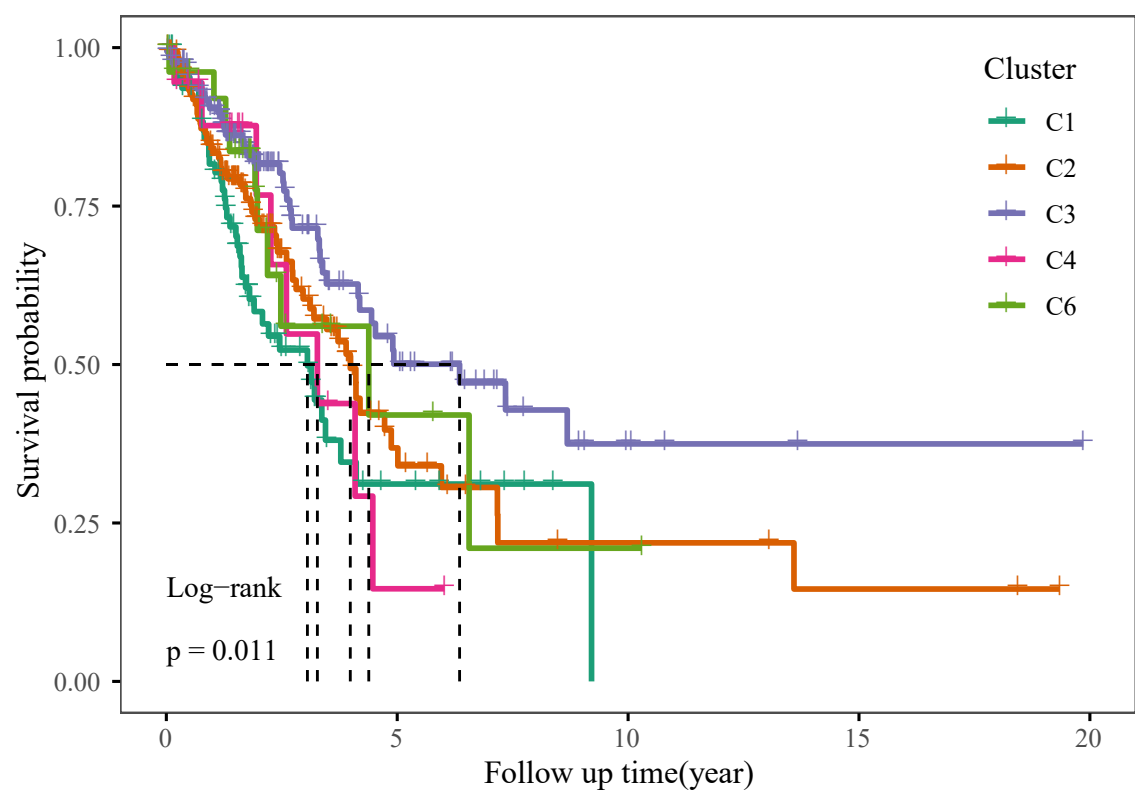

### Number at risk

|    | 0   | 5  | 10 | 15 | 20 |
|----|-----|----|----|----|----|
| C1 | 81  | 7  | 0  | 0  | 0  |
| C2 | 141 | 13 | 4  | 2  | 0  |
| C3 | 175 | 23 | 4  | 1  | 0  |
| C4 | 18  | 1  | 0  | 0  | 0  |
| C6 | 27  | 3  | 1  | 0  | 0  |

Follow up time(year)

Supplement: Supplementary Materials — Figure S1: KM survival curve of the six published immunoinfiltrating molecular subtypes. Figure S2: immune cell scores (B lineage, cytotoxic lymphocytes, endothelial cells, fibroblasts, monocytic lineage, myeloid dendritic cells, and neutrophils) of each sample. Figure S3: A: with the gradual increase of lambda, the number of independent variable coefficients approaching 0 also increases gradually. B: when lambda = 0.02797, the model reached the optimal value. Figure S4: the expression of five genes made a significant prognosis difference between the risk of high and low expression in the sample. Figure S5: the differences of our models in the chemotherapy and radiotherapy samples. Table S1: The sample clinical information of databases. [file 8832739.f1.zip › Figure S1.pdf]

Cluster 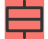 C1 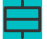 C2

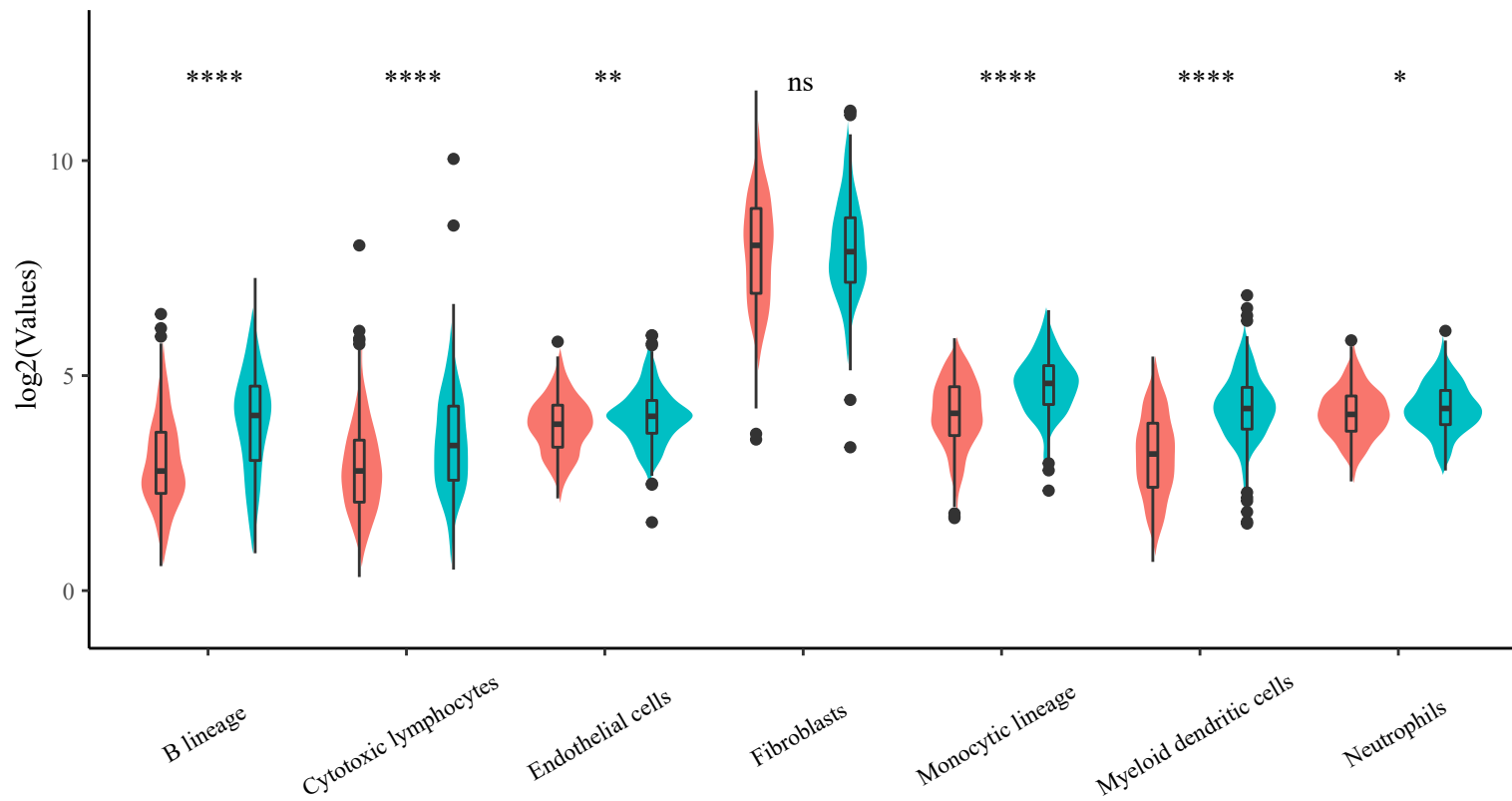

Supplement: Supplementary Materials — Figure S1: KM survival curve of the six published immunoinfiltrating molecular subtypes. Figure S2: immune cell scores (B lineage, cytotoxic lymphocytes, endothelial cells, fibroblasts, monocytic lineage, myeloid dendritic cells, and neutrophils) of each sample. Figure S3: A: with the gradual increase of lambda, the number of independent variable coefficients approaching 0 also increases gradually. B: when lambda = 0.02797, the model reached the optimal value. Figure S4: the expression of five genes made a significant prognosis difference between the risk of high and low expression in the sample. Figure S5: the differences of our models in the chemotherapy and radiotherapy samples. Table S1: The sample clinical information of databases. [file 8832739.f1.zip › Figure S2.pdf]

A

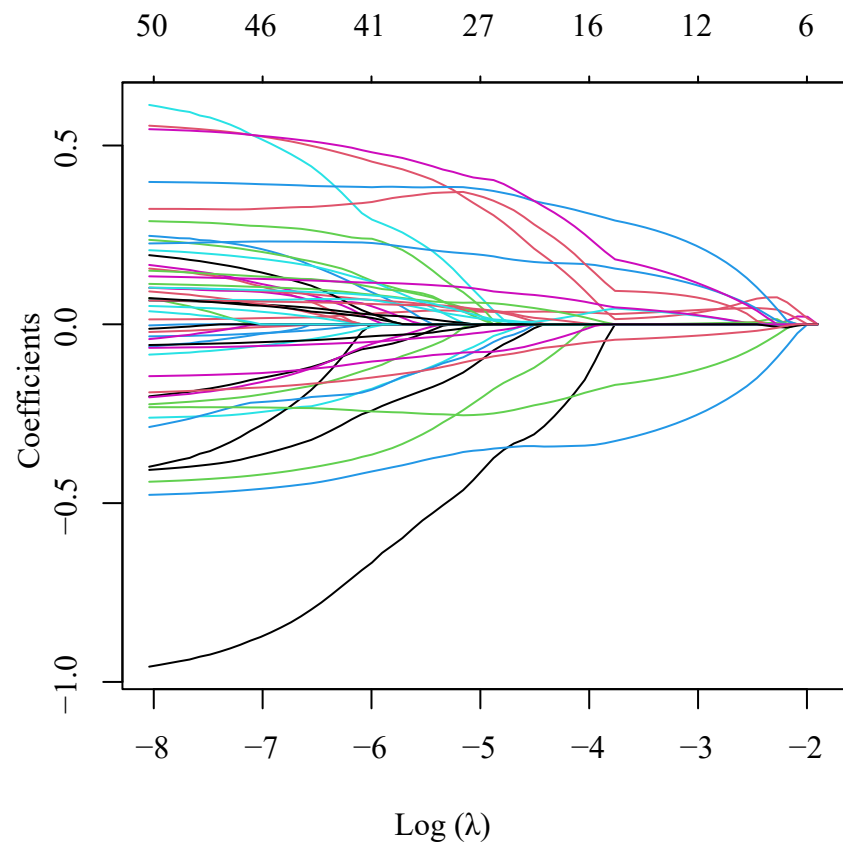

B

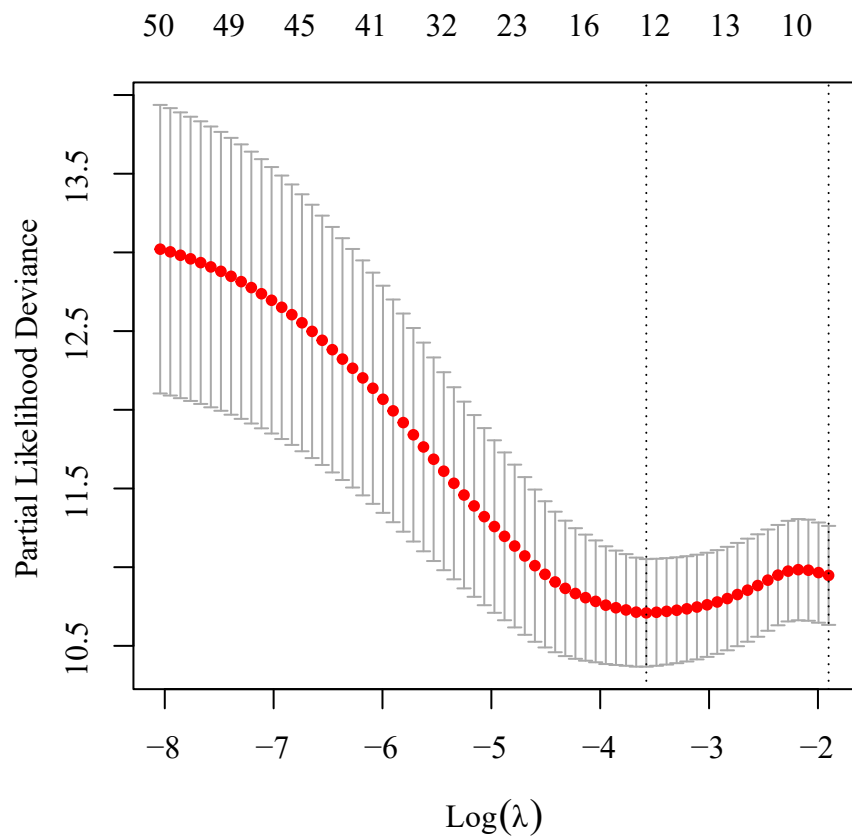

Supplement: Supplementary Materials — Figure S1: KM survival curve of the six published immunoinfiltrating molecular subtypes. Figure S2: immune cell scores (B lineage, cytotoxic lymphocytes, endothelial cells, fibroblasts, monocytic lineage, myeloid dendritic cells, and neutrophils) of each sample. Figure S3: A: with the gradual increase of lambda, the number of independent variable coefficients approaching 0 also increases gradually. B: when lambda = 0.02797, the model reached the optimal value. Figure S4: the expression of five genes made a significant prognosis difference between the risk of high and low expression in the sample. Figure S5: the differences of our models in the chemotherapy and radiotherapy samples. Table S1: The sample clinical information of databases. [file 8832739.f1.zip › Figure S3.pdf]

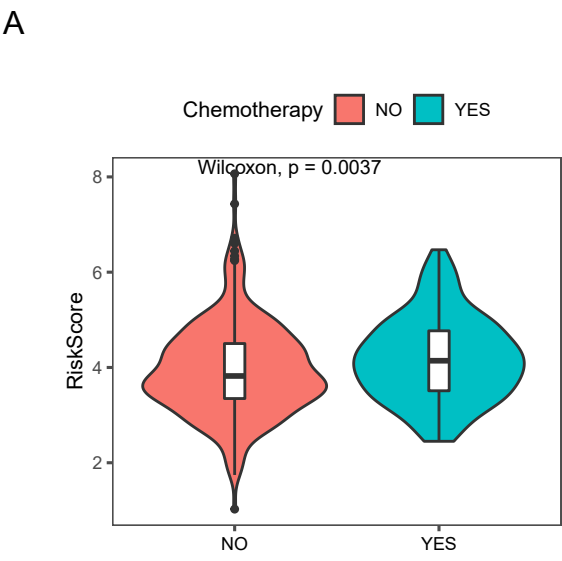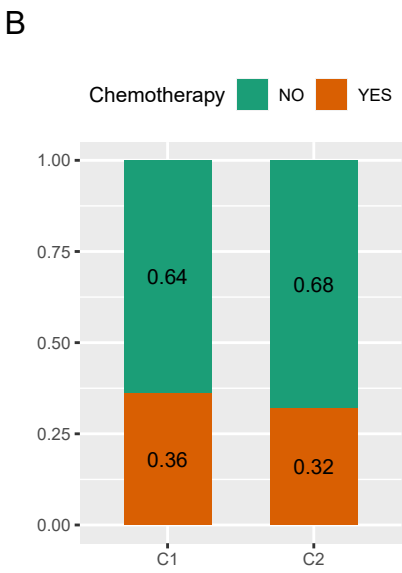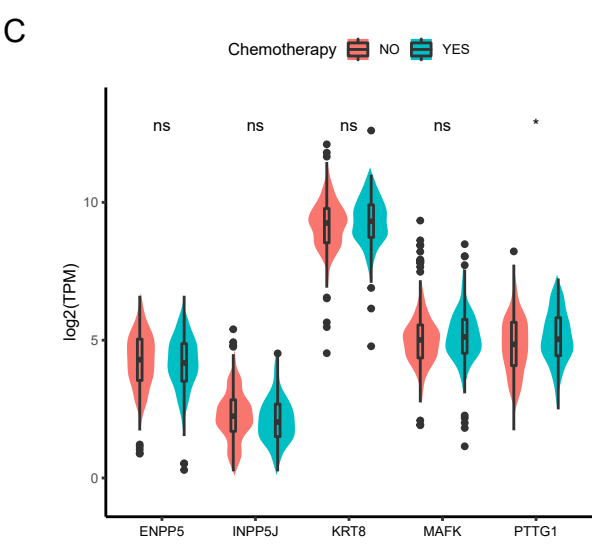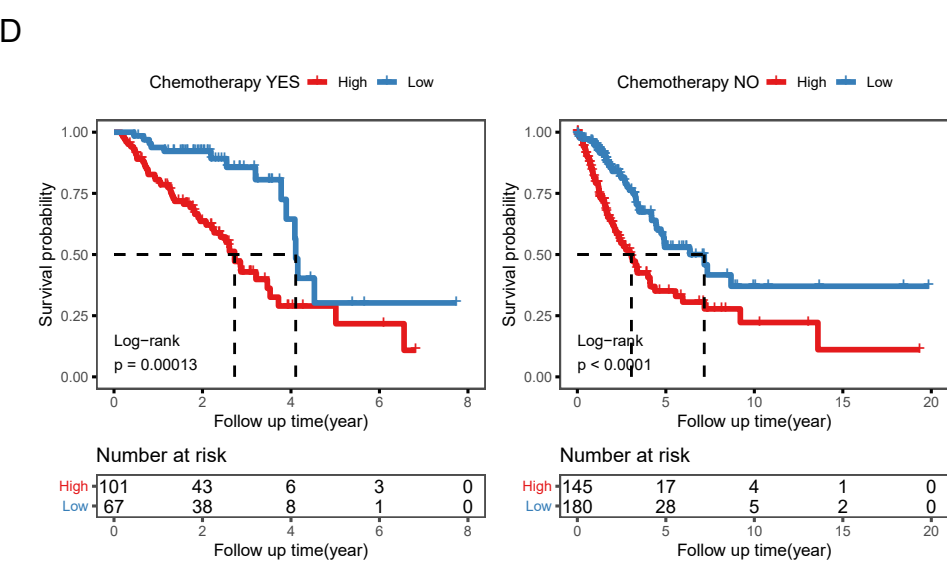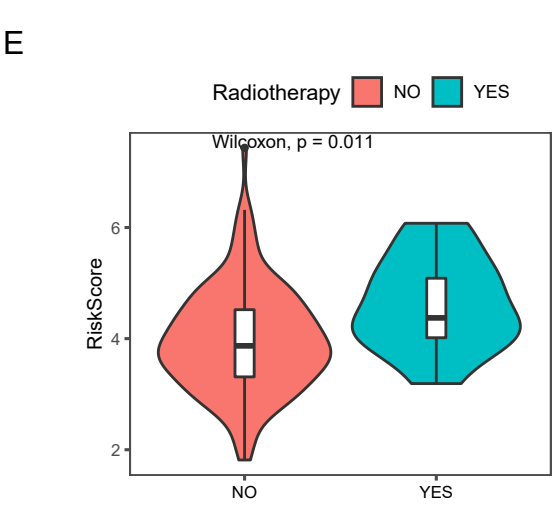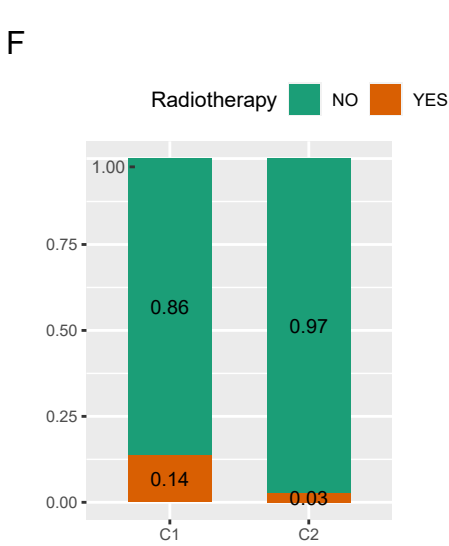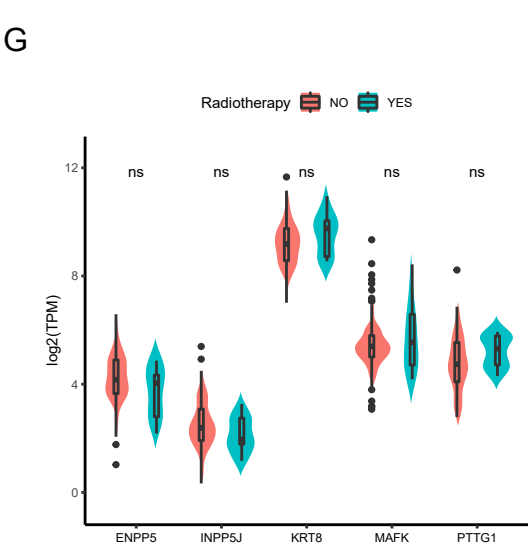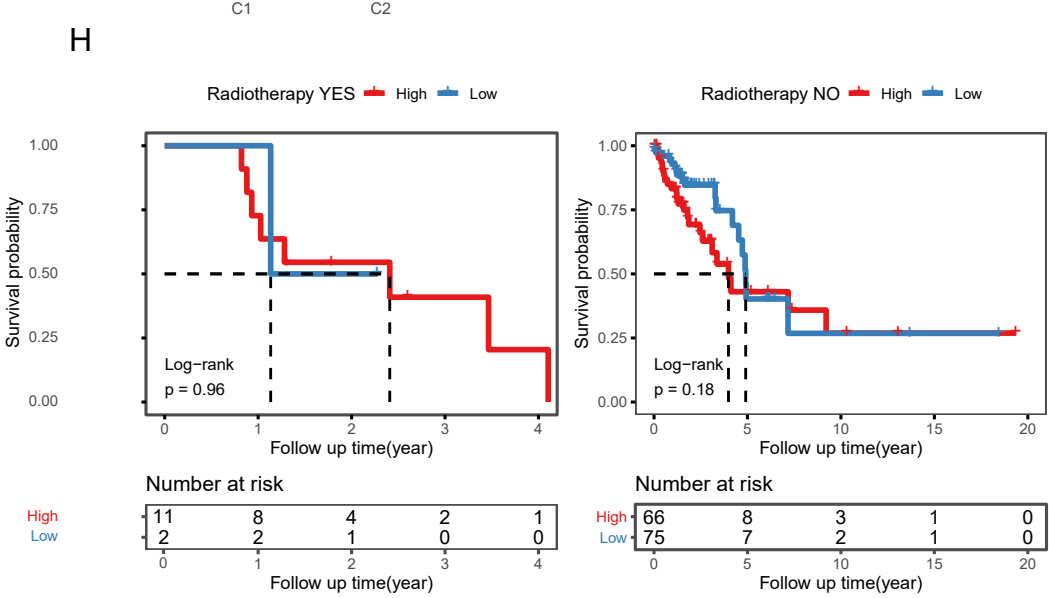

Supplement: Supplementary Materials — Figure S1: KM survival curve of the six published immunoinfiltrating molecular subtypes. Figure S2: immune cell scores (B lineage, cytotoxic lymphocytes, endothelial cells, fibroblasts, monocytic lineage, myeloid dendritic cells, and neutrophils) of each sample. Figure S3: A: with the gradual increase of lambda, the number of independent variable coefficients approaching 0 also increases gradually. B: when lambda = 0.02797, the model reached the optimal value. Figure S4: the expression of five genes made a significant prognosis difference between the risk of high and low expression in the sample. Figure S5: the differences of our models in the chemotherapy and radiotherapy samples. Table S1: The sample clinical information of databases. [file 8832739.f1.zip › Figure S5.pdf]
